# The shape-dependent surface oxidation of 2D ultrathin Mo_2_C crystals†

**DOI:** 10.1039/c9na00504h

**Published:** 2019-11-11

**Authors:** Lin Li, Min Gao, Jonas Baltrusaitis, Dong Shi

**Affiliations:** School of Optoelectronic Science and Engineering, University of Electronic Science and Technology of China Chengdu 610054 P. R. China dshi@uestc.edu.cn; School of Electronic Science and Engineering, University of Electronic Science and Technology of China Chengdu 610054 China; Department of Chemical and Biomolecular Engineering, Lehigh University 111 Research drive Bethlehem PA 18015 USA

## Abstract

2D atomic crystals have been widely explored, usually owing to their numerous shapes, of which the typical hexagon has drawn the most interest. However, the relationship between shape and properties has not been fully probed, owing to the lack of a proper system. Here, we demonstrate for the first time the shape-dependent surface oxidation of 2D Mo_2_C crystals, where the elongated flakes are preferentially oxidized under ambient conditions when compared with regular ones, showing higher chemical activity. The gradual surface oxidation of elongated Mo_2_C crystals as a function of time is clearly observable. Structural determinations reveal that a discrepancy in the arrangement of Mo and C atoms between elongated and regular crystals accounts for the selective oxidation behavior. The identification of the shape-dependent surface oxidization of Mo_2_C crystals provides significant possibilities for tuning the properties of 2D materials *via* shape-control.

## Abstract 2

The shape-dependent surface oxidation of 2D ultrathin Mo_2_C crystals is demonstrated.

## Graphical abstract

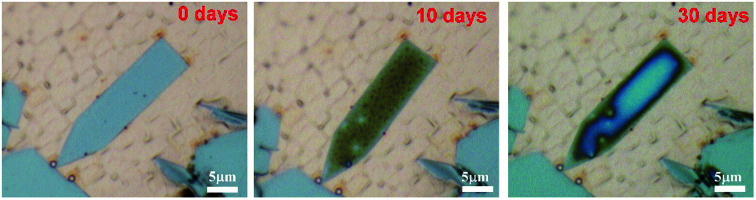

In the past few years, the field of 2D materials has witnessed an explosive development, with novel materials produced, and unusual properties demonstrated.^1–4^ Of those newly-developed 2D materials, transition metal dichalcogenides (TMDs) and several elemental analogues of graphene have drawn considerable attention because of their exotic electronic properties.^5–8^ Recently, as a new member of this family, transition metal carbides (TMCs) have emerged,^9–11^ which combine the properties of metals and covalent compounds, owing to their good mechanical stability and high electrical and thermal conductivity. On the other hand, it is well known that atomic crystals usually have numerous shapes, of which the hexagon is the most widely explored, as in graphene.^12–14^ Intensive efforts, then, have been devoted to the shape control of 2D crystals, aiming to realize property tuning. It has been evidenced that edge structure varies for different shaped graphene.^15^ However, a clear and identified relationship between the shape and properties of 2D materials has still not been demonstrated till now, mainly owing to the lack of a proper system. In a very recent work, Mo_2_C crystals with several regular shapes were simultaneously prepared in one pot during a chemical vapor deposition (CVD) process,^16–19^ thus offering an ideal system for such a purpose.

Herein, we present for the first time a shape-dependent surface activity of Mo_2_C crystals. Both regular and elongated crystals are produced on a liquid Cu surface, showing a distinctively selective surface oxidation. It is demonstrated that the elongated crystals are more easily oxidized while those regular shaped crystals remain stable without surface change. The selective oxidation study is applied to all the shaped crystals, including the elongated square, pentagonal and hexagonal. Real-time surface evolution over 30 days is studied, offering direct evidence. Further TEM characterization shows the striking discrepancy in atomic arrangement, accounting for the selective features. The selective surface oxidation of elongated Mo_2_C crystals can served as an effective technique for structural identification, offering an example of shape-property characteristics.

Sample preparation was conducted by using ambient pressure CVD. Liquid Cu^20,21^ was employed as the catalyst on the supporting Mo foil, while methane (CH_4_) was used as the carbon precursor, and hydrogen (H_2_) was used as the reduction and carrier gas. The whole growth process is clearly schematized in Fig. 1a. Fig. 1b shows an optical image of the as-grown samples, wherein large-area Mo_2_C flakes are uniformly dispersed on the whole Cu surface. It can be seen that the typical hexagonal flakes are present in the highest percentage, while other shaped flakes can also be observed; of particular interest is the appearance of elongated square ones, as highlighted by arrows in Fig. 1b. Strictly speaking, Mo_2_C is not categorized as a layered material, since Mo and C are covalently bonded within the structure (Fig. 1c inset). To probe the structure, X-ray photoelectron spectroscopy (XPS) measurements have been performed. It is obviously found that there exist two typical peaks around 230 eV and 280 eV (Fig. 1c), correlated with the elements Mo and C, respectively. For the two signals, high-resolution peak analysis is displayed in Fig. 1d and e. Based on the measurements, it is indicated that the crystal is composed of Mo and C with an atomic ratio of ∼2 : 1, consistent with the previous reports.^22,23^ The samples on a Cu surface were further examined by using X-ray diffraction (XRD). As shown in Fig. 1f, the identified peaks labelled with squares are assigned to a pure monoclinic Mo_2_C phase (JCPDS card no. 15-0457) (Fig. S1†), while the peaks marked with triangles derive from the Cu substrate.^24,25^ It has been reported that Mo_2_C has high stability in inert gas or reduction gas. In our case, XRD measurements have been conducted to probe the stability as a function of temperature (Fig. S2†). The characteristic peaks could be clearly observed and remained unchanged, suggesting the structure remained the same upon changes in temperature. A transferring process was performed, where a wet etching method enables the large-area crystals to smoothly transfer onto SiO_2_/Si (further details are provided in the ESI†). Atomic force microscopy measurements show that the thickness of those crystals falls into a range of around several nanometers (Fig. S3†). However, the shape of the Mo_2_C crystals might be determined by several growth factors, posing a great challenge in precisely fabricating one specific shape. However, the variation in shape provides a proper platform to study the discrepancy in properties among all those differently shaped crystals.

**Fig. 1:**
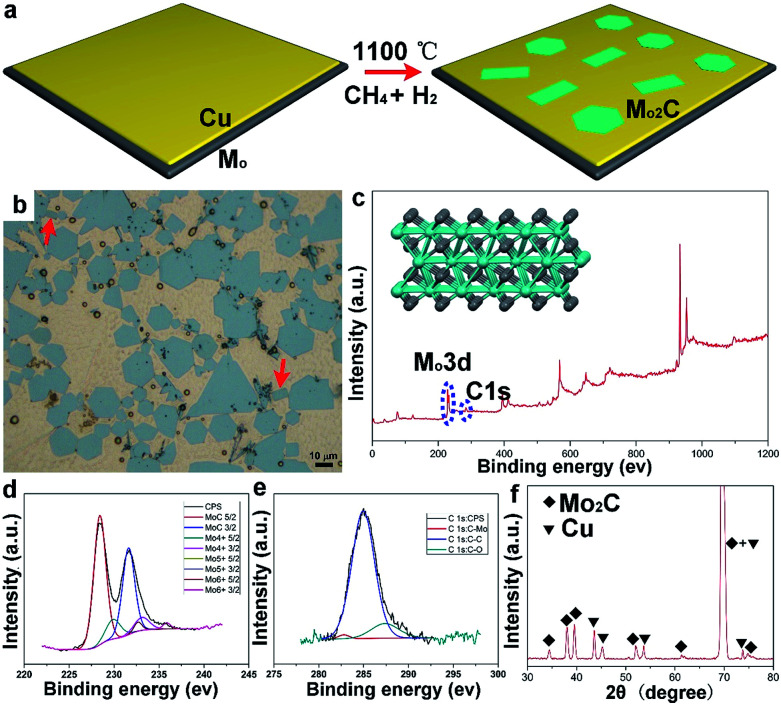
The growth of Mo_2_C crystals by the CVD method. (a) A schematic diagram of the growth process on a liquid Cu surface. (b) Optical images of large-area Mo_2_C flakes; note that elongated flakes are highlighted with arrows. (c) XPS survey spectrum of Mo_2_C crystals. (d and e) High-resolution spectra of Mo 3d and C 1s, confirming the existence of Mo_2_C. (f) XRD patterns of Mo_2_C on the Cu substrate; the assigned peaks of Mo_2_C and Cu are labelled, respectively.

After the CVD growth, the chemical activity of Mo_2_C crystals with different shapes was probed. In this case, the flow rate of CH_4_ was modulated to achieve a higher percentage of elongated flakes (Fig. S4†). By decreasing the carbon concentration, we found that more elongated square flakes are produced when comparing with the case in Fig. 1b. A statistical analysis was performed among all the flakes, where the elongated flakes were found to account for nearly 40 percent of the flakes (Fig. 2a). All the flakes share an average size of 11 ± 1 μm (Fig. 2b), suggesting a uniform growth in size. After exposure to air for 5 days, the elongated flakes were found to experience a distinct change, as shown in Fig. 2c. The surface of the elongated shaped flakes became dark when compared with those of the regular hexagonal ones. It is worth noting that the surface evolution is highly related to the time. Fig. 2c–f demonstrate a clear evolution of large-area elongated flakes as a function of time, with 5 d, 15 d, 25 d and 30 d, respectively. Note that the optical images are not collected from the same area. It can be clearly seen that the color of the elongated flakes becomes more and more intense, indicating a thicker and thicker surface oxidation, while the neighboring hexagonal flakes highlighted by arrows remain unchanged (Fig. 2f). We also noticed that even with a much longer time of up to 60 days, the regular hexagonal flakes still remained the same without surface oxidation. In another word, chemical activity is varied between the elongated and regular crystals, where those transformed flakes are more active. Based on the observations, the surface changes of the flakes are found to follow an obvious shape-dependent selectivity.

**Fig. 2:**
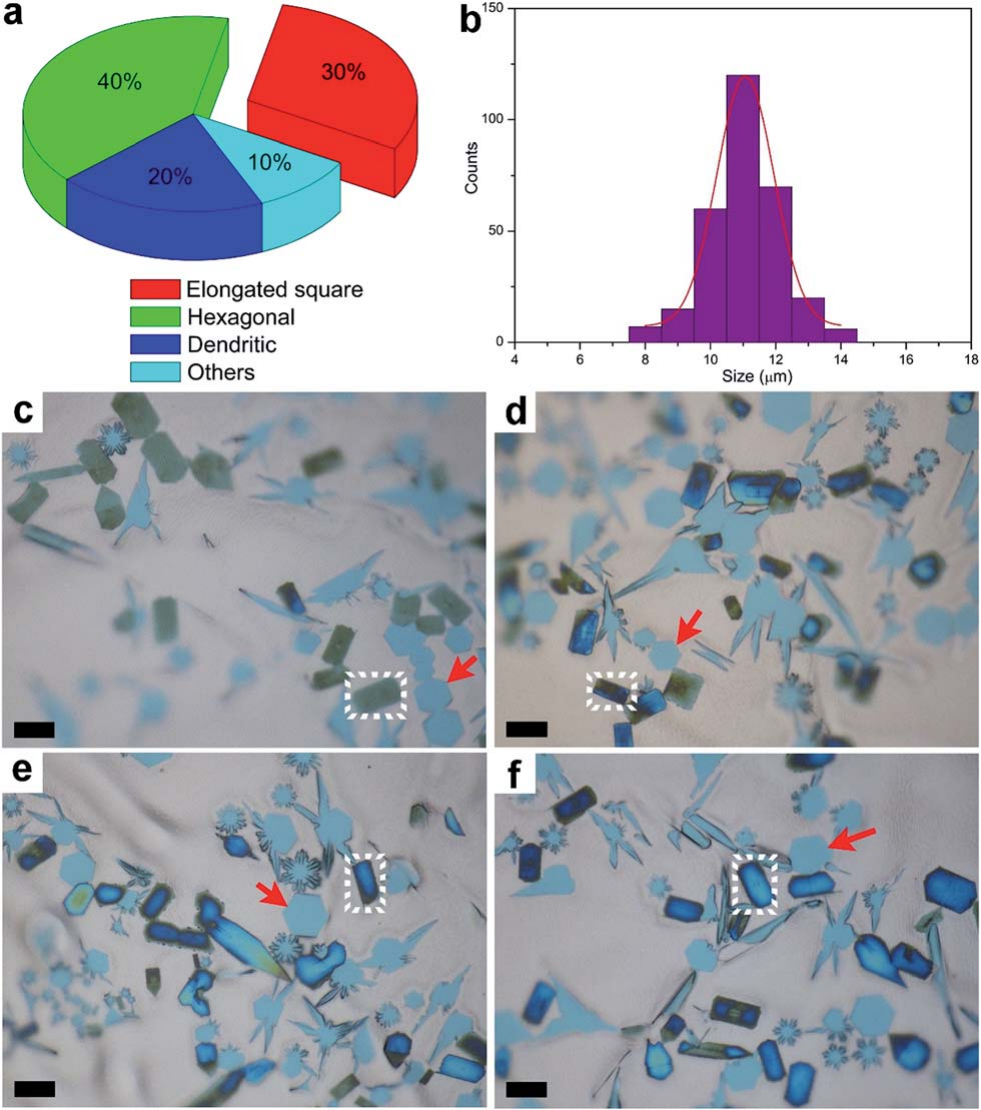
Surface evolution of the elongated square Mo_2_C flakes on the liquid Cu surface. (a) Relative percentages of the different shaped crystals across the whole surface. (b) Size distribution of Mo_2_C flakes on the whole surface. (c–f) The gradual surface oxidation of the elongated Mo_2_C flakes as a function of time, with 5, 15, 25 and 30 days as a sequence. The changed elongated flakes are highlighted in white and the unchanged flakes with regular hexagonal shape are highlighted with red arrows. All the scale bars are 10 μm.

Besides the elongated square shaped flakes, the surface evolution is also possible for other elongated flakes, as clearly show in Fig. 3 and S5.† It is found that the flake is derived from a regular pentagonal flake, in which the lateral direction is extended. The elongated pentagonal flake is found to have a similar surface behavior, and a gradual evolution profile of one flake as a function of time is fully exhibited in Fig. 3a–g. In this case, we traced the change and found that it took about 30 days to progress from newly grown to fully covered. Such a surface change of the elongated shaped flakes was found to be very common over the whole surface (Fig. S6†). To further probe the surface change, Raman spectra of the elongated shaped flakes before and after surface oxidation are shown in Fig. 3h and i. It was found that the elongated pentagonal flake exhibited a striking difference; the typical Raman peaks around 650 cm^−1^ suggested the existence of Mo_2_C crystals,^26^ while the newly emerged signals with stronger intensity derived from the changed sample might be correlated with molybdenum oxides (Fig. S7†).^27^ Based on a previous report^28^ and our Raman spectrum, it was deduced that the two peaks at the positions of 820 cm^−1^ and 996 cm^−1^ are typically characteristic of MoO_*x*_ and the detailed surface information can be seen in Fig. S8.†

**Fig. 3:**
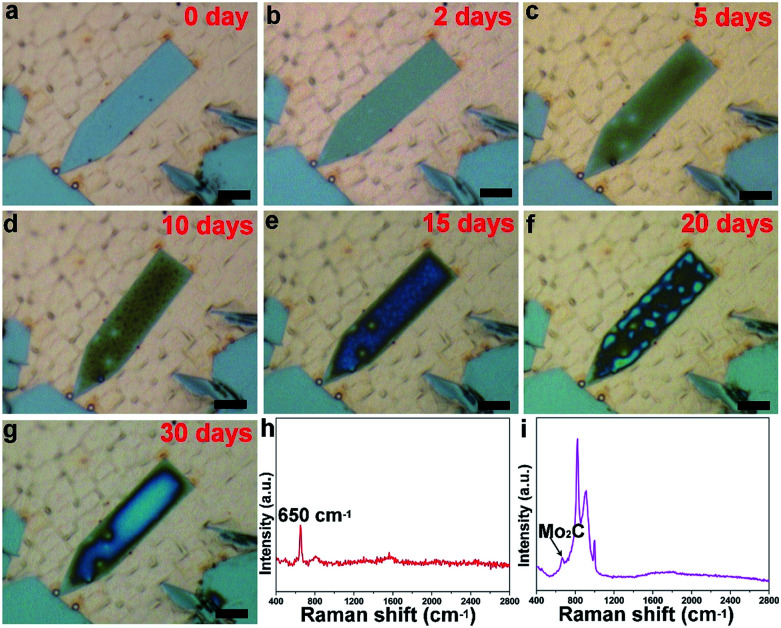
Surface oxidation of a single elongated pentagonal Mo_2_C flake. (a) Freshly grown elongated flake. (b–g) Optical images of the elongated flake with gradual surface evolution under ambient conditions. (h and i) Typical Raman spectra of elongated flakes before and after surface oxidation, corresponding to the flakes in (a) and (g), respectively. All the scale bars are 5 μm.

To disclose the changing process, scanning electron microscopy (SEM) and energy dispersive spectroscopy (EDS) measurements have been performed. It can be seen that the elongated pentagonal flake exhibits much darker color contrast (Fig. 4a) than the regular ones, indicating more oxidation. The high-magnification image shows the existence of aggregated particles covering the dark area of the surface (Fig. 4b). Further EDS characterization (Fig. 4c) revealed an O peak with a high ratio besides the intrinsic elements of Mo and C. On this basis, the oxidation was deduced to form molybdenum oxides. It is reasonable to conclude that the elongated crystals have a much higher activity, leading to the easier oxidation. The composition and surface chemical state of the as-oxidized Mo_2_C hybrid were further investigated by using X-ray photoelectron spectroscopy (XPS) and all binding energy (BE) values were calibrated using the C 1s peak at 284.6 eV as a reference. In our work, after synthesis and reduction, only the signals of Mo and C were detected, indicating the stability of the as-grown samples. As regards the point of a surface terminating layer, it is suggested that the layer is very thin when compared to the thick Mo_2_C layers. Thus, it might be negligible in the whole profile of the samples. As shown in Fig. S9 and S10,† the survey spectrum of Mo_2_C shows distinct signals at 231.5, 285.1, and 531.5 eV, which can be assigned to Mo 3d, C 1s, and O 1s, respectively. Fig. 4d shows the high resolution XPS spectrum of Mo 3d, which shows two major peaks with binding energies of 228.6 and 231.9 eV, and two weak peaks at 229.3 and 234.7 eV. The former two peaks can be ascribed to Mo 3d of Mo_2_C and the latter two peaks can be ascribed to oxidized molybdenum with various oxidation states (MoO_*x*_). For the carbon high resolution XPS spectrum (Fig. 4e), the peak at a binding energy of 284.2 eV can be assigned to the molybdenum-bonded carbon, whereas those at 285.9 and 288.6 eV can be ascribed to the carbons in non-oxygenated C=O, and O–C=O, respectively. Finally, the O 1s signal shows one peak (530.5 eV) belonging to the Mo–O bonds, and another one (533.2 eV) belonging to C=O, respectively (Fig. 4f).

**Fig. 4:**
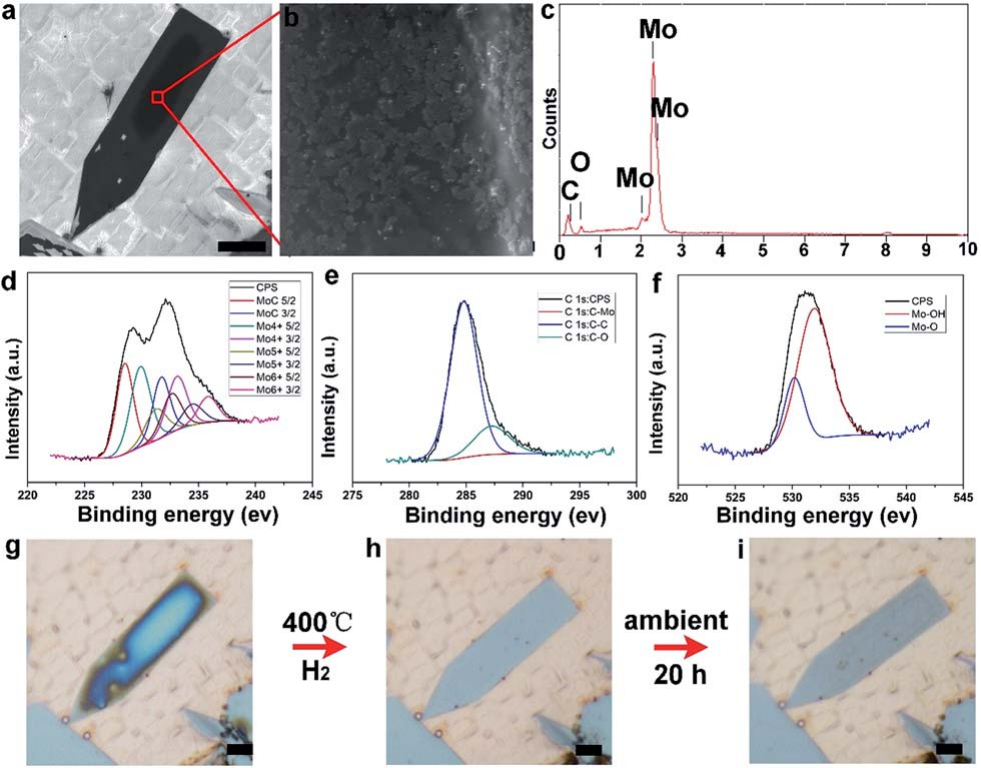
Characterization of surface oxidation of an elongated flake. (a) SEM images of the elongated flake, on which an obvious black area can be observed. (b) High-magnification image of the labeled area in (a), showing dispersed nano-clusters aggregated on the surface. (c) EDS spectrum of the as-oxidized flake, from which the element O with a high percentage was detected, besides the intrinsic elements of Mo and C. (d–f) High-resolution XPS spectra of Mo_2_C after oxidation, showing the Mo 3d, C 1s and O 1s signals, respectively. (g–i) The reduction of the as-oxidized elongated flake under a hydrogen atmosphere at 400 °C and further re-oxidization under ambient conditions, displaying an oxidization cycle. The scale-bars in (a, g, h, and i) are 5 μm and in (b), it is 100 nm.

To verify the presence of molybdenum oxides, we have conducted reduction experiments, in which temperature was set at 400 °C in pure H_2_ with a flow rate of 200 sccm for 2 h. It can clearly be observed that the high level of oxidation on the surface of the elongated Mo_2_C flakes (Fig. 4g) gradually disappears after the H_2_ reduction (Fig. 4h), suggesting the presence of as-formed molybdenum oxides. Furthermore, the as-reduced elongated Mo_2_C flake would experience the same oxidation process again when re-exposed to the air (Fig. 4i). It should be noted that this behavior is also possible for the other elongated Mo_2_C flakes (Fig. S11†), further indicating a general guideline among all the shaped flakes. Regarding the surface oxidation behavior of the as-grown Mo_2_C flakes, we performed a statistical analysis of their shapes. It was found that all the elongated flakes share the easy oxidation feature, including elongated square, pentagonal, and hexagonal cases (Fig. S12†). It was well confirmed that all the three kinds of flakes are derived from regular shapes, extended along two lateral directions (Fig. S13†). During the growth step, different crystal planes experience discrepancies in growth rate, highly related with energy, where those planes with a higher energy grow faster and thus result in more stable formed planes with lower energy. Based on this proposal, it was deduced that the shape-dependent oxidation process of Mo_2_C is tightly related with the inner crystal structures. In our experiments, it was found that the shape is rather sensitive to the CVD growth conditions, especially the flow rate of CH_4_ (Fig. S14†). In this case, for Mo_2_C, it was deduced that the crystal growth operates in the regime of carbon supersaturation where the dominant mechanism of growth is through 2D nucleation on the crystal face. Thus, the relative growth rate of the different crystallographic faces changes with supersaturation, whereby different crystal morphologies can be obtained by tuning the flow rate of the CH_4_.

To further disclose the structure of those elongated flakes, TEM measurement has been conducted. Selected-area electron diffraction (SAED) patterns of the regular flakes are presented in Fig. 5a, while the patterns of the elongated ones are displayed in Fig. 5d. Notably, the SAED patterns of the regular and elongated crystals exhibit differences, where extra superlattice spots are observed in the pattern of the triangular case. To reveal the origin of this difference, a model of the Mo_2_C crystal structure was constructed and its calculated SAED patterns are shown in Fig. 5b and e. A different false color is utilized to label certain set of spots sharing the same *d*-spacing, and the detailed *d*-spacing data are listed in Table S1.† Comparing the calculated SAED pattern with experimentally obtained data, we can conclude that in the regular crystal, Mo atoms follow a close-packed stacking in the (001) plane and they form an *AB* stacking along the [001] direction, while carbon atoms randomly fill half of the octahedral vacancies created by the Mo planes (Fig. 5c). However, obviously, in the elongated crystal, additional sets of spots can be observed, and these extra spots can be ascribed to long range carbon vacancy ordering schemes along either the [100] or [010] direction (Fig. S15†). Specifically, carbon atoms alternately occupy the octahedral vacancies along the [100] or [010] direction and a unique staggered configuration is formed (Fig. 5f and S16†). Based on the observations and discussion, it is reasonable to deduced that the C coordination number of the metal atoms in the elongated crystals is lower than that of the regular ones. Previous theoretical studies suggested that the chemical activity of carbides becomes much higher with a decreased C coordination number,^29–31^ consistent with our results. The TEM results clearly suggest that Mo_2_C crystals with different shapes exhibit discrepancies in carbon coordination number, which can be manipulated during growth, offering the possibility for tuning properties.

**Fig. 5:**
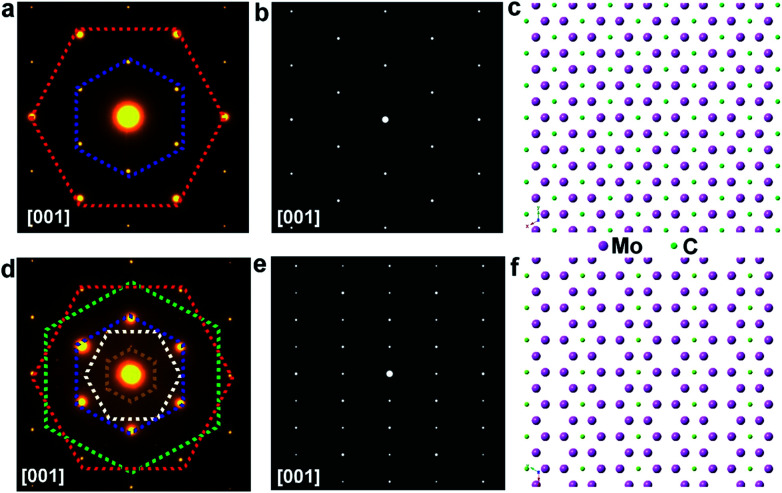
TEM structural characterization of regular and elongated Mo_2_C flakes. (a and d) SAED patterns of regular and elongated Mo_2_C crystals, where each labelled pattern corresponds to each atomic orientation. (b and e) Corresponding calculated SAED patterns. It is noted that the calculated model is highly consistent with the experimental data. (c and f) Atomic models of the regular and elongated crystals, where, for the elongated Mo_2_C crystal, random vacant sites are present, which might be highly related to the higher activity of the surfaces.

The proposed atomic model of Fig. 5c is the commonly seen alpha phase Mo_2_C (space group *P*3̄*m*1). The close-packed Mo–C–Mo atomic planes in one unit length form an intralayer *abc* stacking sequence. On the other hand, the diffraction pattern obtained from the elongated crystal shows clear superspots compared to the conventional alpha phase Mo_2_C. The superspots originate from the presence of periodic carbon vacancies that have been widely seen in many other metal carbides. The proposed geometric pattern of carbon vacancies shows a periodic stripe structure, and the presence of periodic carbon vacancies renders the emergence of rectangular-like 1 × 2 superstructures. It is known that Mo_2_C shows an in-plane 6-fold symmetry, thereby the stripe-like carbon vacancies could exist in 3 degenerate (every 120°) crystal orientations leading to the formation of the 2 × 2 superstructures shown in the experimental result. It can be seen in the experimental results that the intensity of the primary diffraction spots is much stronger than that of the superspots. On the other hand, the intensity of the primary spots and superspots is roughly equivalent in the simulated image. Hence, we can conclude that the main matrix of elongated crystal maintains the alpha phase Mo_2_C; however, the surface or a small portion of the bulk crystals contains the proposed short-range periodic carbon vacancies. Given the complicated structures of the as-grown Mo_2_C, there might be another possibility whereby the structures that are oxidation inactive probably arise from carburization or they are over carburized. Structurally, it is known that the carburization rate and degree (Mo_2_C_*x*_) are condition dependent.^32,33^ The CVD method can probably prepare different Mo_2_C_*x*_ under different carburization conditions. The related work is still ongoing.

To summarize, the shape-dependent surface oxidation of CVD grown Mo_2_C crystals has been demonstrated. The gradual surface evolution of elongated flakes as a function of time has been fully investigated, indicating different stabilities between the regular and elongated crystals and showing selectivity. Structural discrepancy between the two kinds of flakes is identified to be responsible for the difference in chemical activity, originating from the arrangements of carbon atoms within the crystal structures. The shape-dependent surface oxidation behavior of Mo_2_C offers a facile and effective method for structural determination and further property control.

## Conflicts of interest

There are no conflicts to declare.

## Supplementary Material

NA-001-C9NA00504H-s001
