# Supplementary material for: The shape-dependent surface oxidation of 2D ultrathin Mo2C crystals
Source: Nanoscale Adv. 2019 Nov 11;1(12):4692–6. doi: 10.1039/c9na00504h (PMC9419211; doi:10.1039/c9na00504h)
Supplement: NA-001-C9NA00504H-s001 [file NA-001-C9NA00504H-s001.pdf]

Electronic Supplementary Material (ESI) for Nanoscale Advances.
This journal is © The Royal Society of Chemistry 2019

Shape-dependent surface oxidation of 2D ultrathin Mo_2C crystals

Lin Li,[1] Min Gao,[2] Jonas Baltrusaitis,[3] Dong Shi[1,*]

[1] School of Optoelectronic Science and Engineering, University of Electronic Science and Technology of China, Chengdu 610054, P. R. China
[2] School of Electronic Science and Engineering, University of Electronic Science and Technology of China, Chengdu 610054, China
[3] Department of Chemical and Biomolecular Engineering, Lehigh University, 111 Research drive, Bethlehem, PA 18015, USA

Correspondence should be addressed to dshi@uestc.edu.cn.

Experimental

Elemental quantification of Mo3d component populations was obtained using XPS combined with Scofield relative sensitivity factors corrected for an electron escape depth. Lorentzian asymmetric peak shape with tail dumping was used in peak fitting. Dumping parameters were set as derived by Baltrusaitis et al.[1]. XPS data processing was performed using a CasaXPS program (suite version 2.3.20). As described by Baltrusaitis et al.[1,2], fitting complex XPS spectral envelopes without prior knowledge of the lineshapes involves certain degree of arbitrariness. It was minimized my constraining Mo3d peak area ratios and their splitting according to the fundamental parameters.

Results

Mo3d5/2 peak can be seen of MoC previously reported at 227.9 eV[3] which is very close to that of metallic Mo[4] due to the existence of Mo-Mo bonds[5]. MoC species comprised about 80% of the total Mo with minor oxygenated MoOx species with varying oxidation states. Oxidized sample has significantly reduced Mo-Mo bond content and increased MoOx species with ~67% of the surface exposed to the oxidized molybdenum compounds. In particular, peaks due to Mo4+, Mo5+ and Mo6+ were detected with the latter particularly significant in the oxidized sample.

Table S1. Calculated d-spacing of different diffraction spots of Mo_2C with and without periodic carbon vacancies.

	(001) plane<br>d-spacing with periodic carobn cacancy (nm)	(001) plane<br>d-spacing without periodic carobn cacancy (nm)
**Orange spots**	0.52	N.A
**White spots**	0.03	N.A
**Blue spots**	0.26	0.26
**Yellow spots**	0.1965	N.A
**Green spots**	0.1733	N.A
**Red spots**	0.1501	0.1501

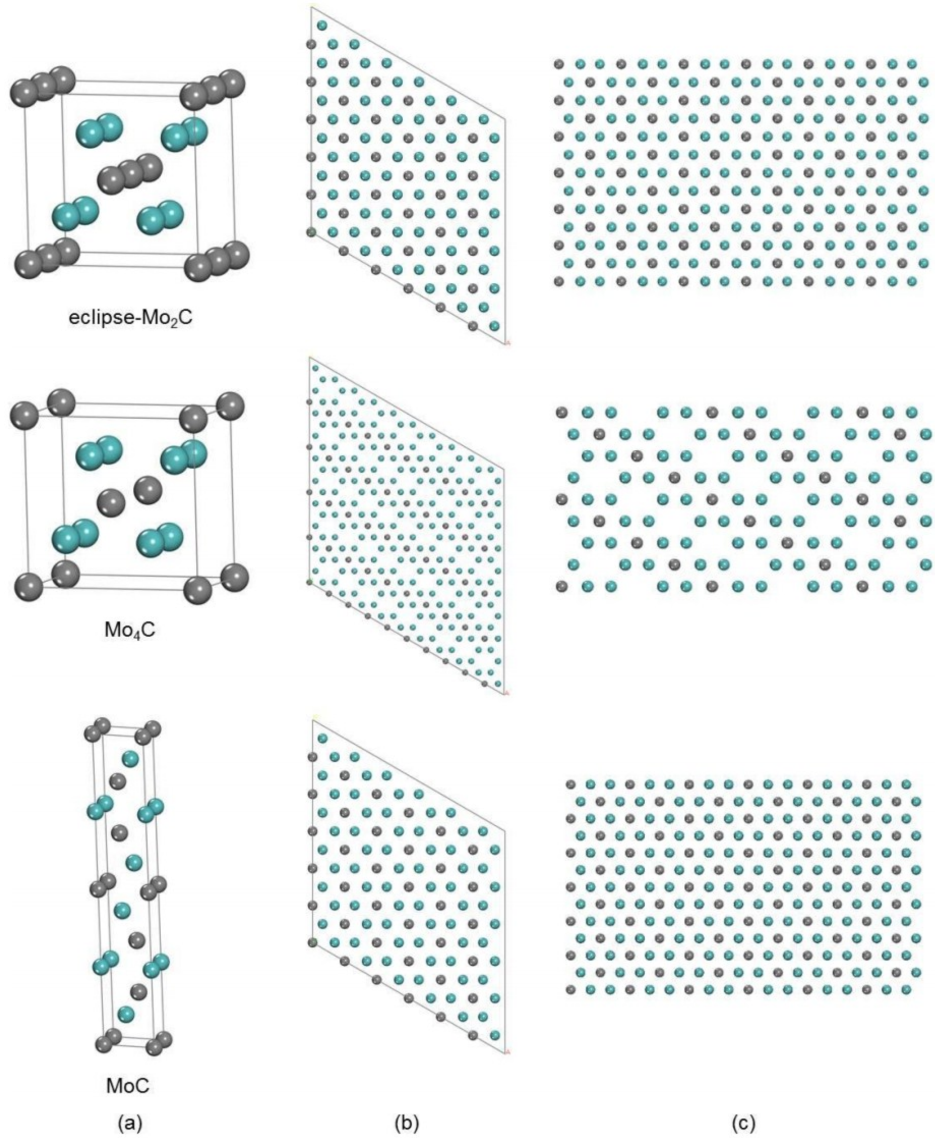

Figure S1. (001) surfaces of eclipse-Mo_2C, Mo_4C and MoC; colum (a) is the cell structures of Mo_2C, Mo_4C based on eclipse- Mo_2C with C in bulk center and ridge center deleted, MoC in P63/MMC space group; colum (b) is the side views of the (001) surfaces; colum (c) is the truncated (001) surfaces for direct comparison with the Figure 5c and 5f in the paper.

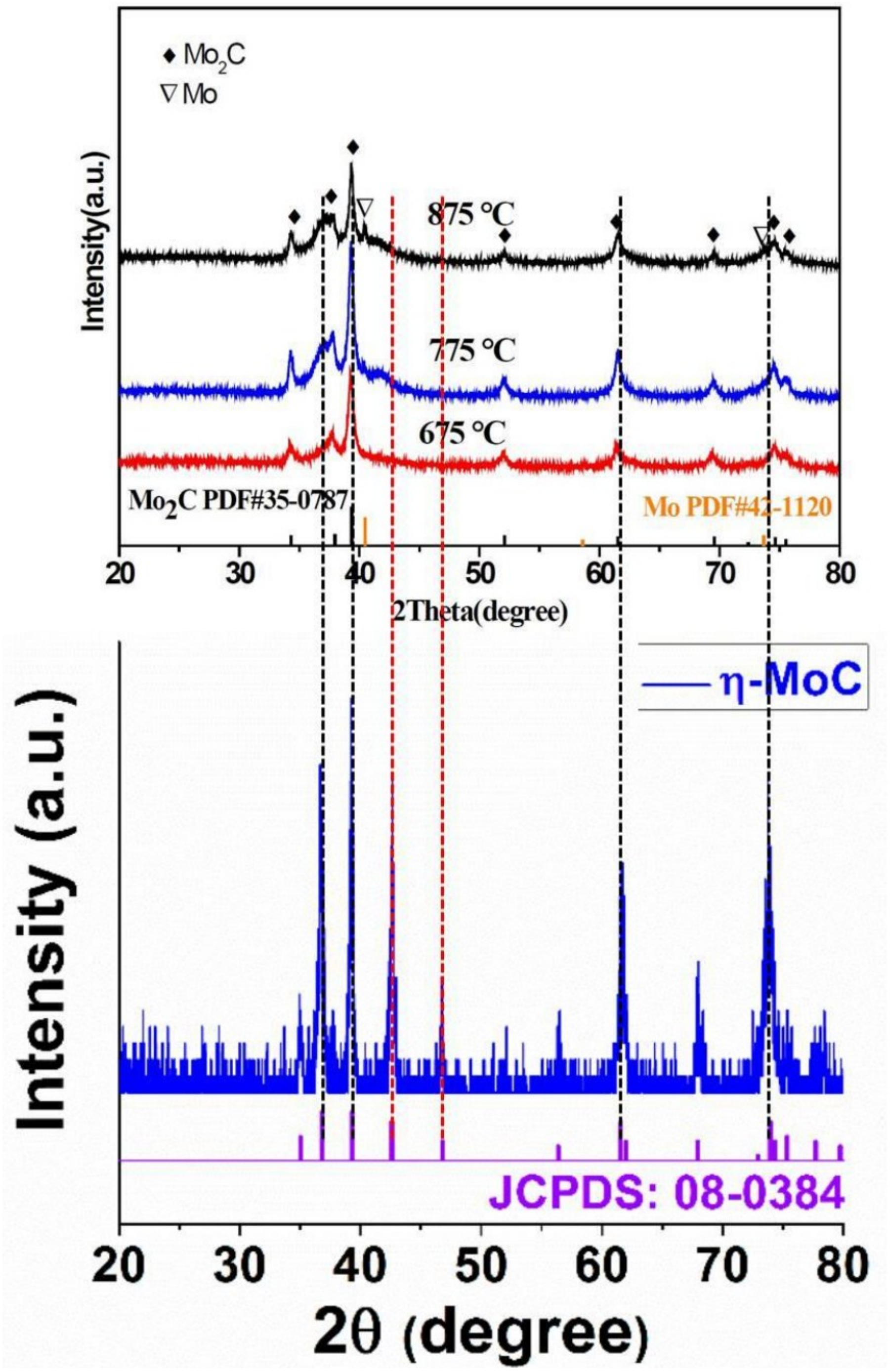

Figure S2. X-ray diffraction (XRD) patterns and standard PDF data of hexagonal β-Mo_2C and η-MoC from experiments.

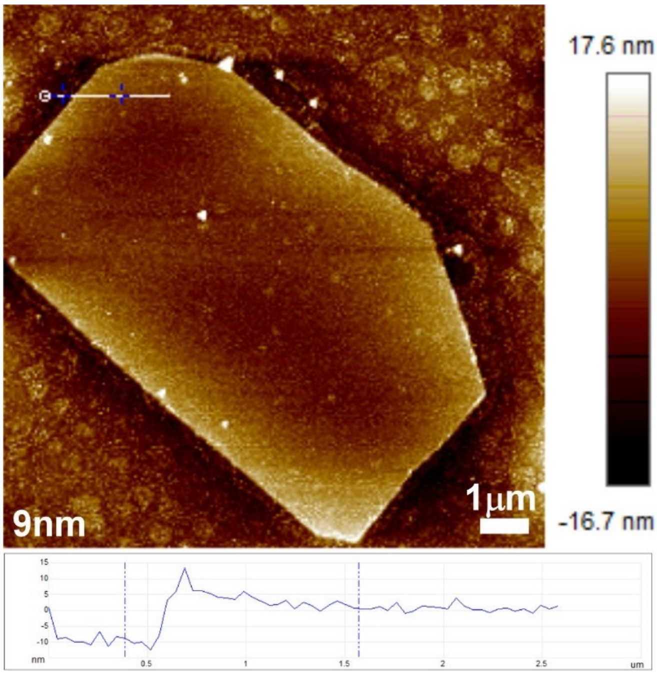

Figure S3. AFM thickness determination of the elongated M_2C.

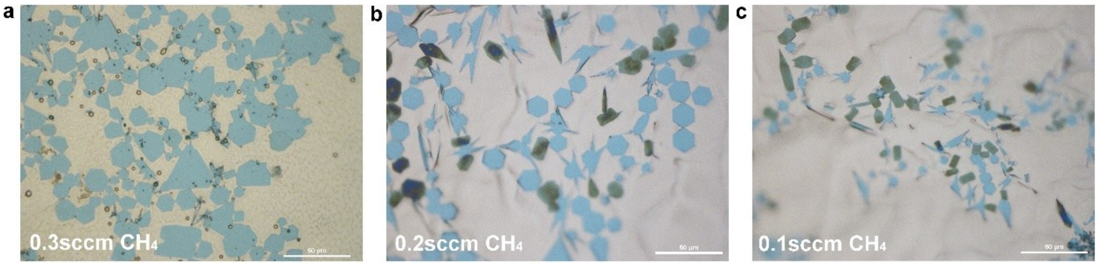

Figure S4. CH_4-controlled morphology evolution of the M_2C crystal on liquid Cu surface by CVD. It is observed that the number of elongated Mo_2C will increase with decrease of the CH_4 gas flow rate.

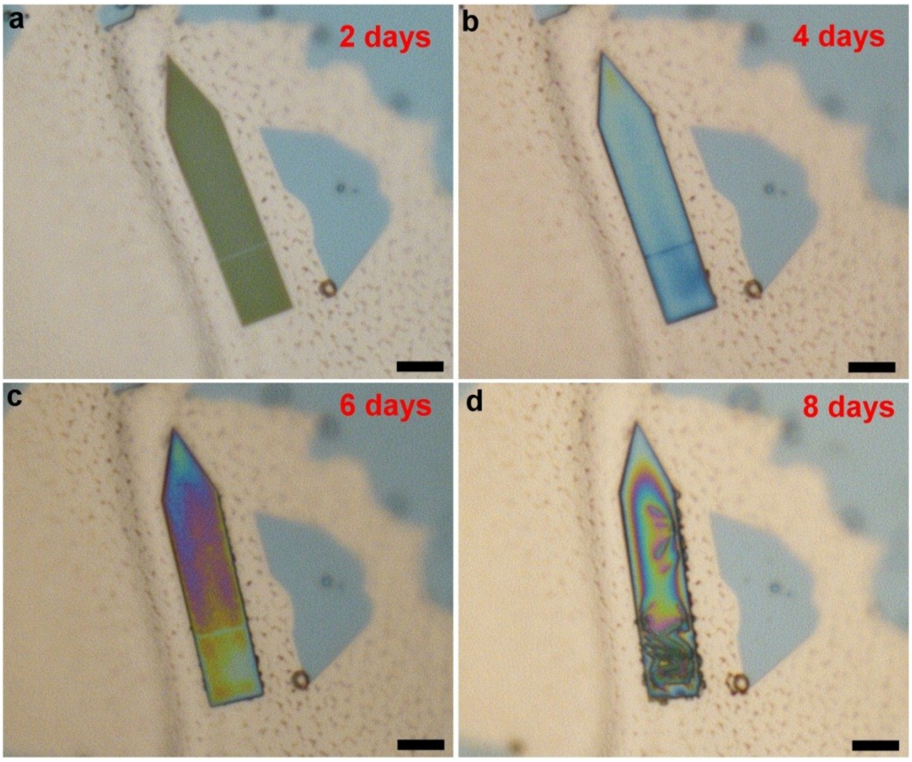

Figure S5. Surface changing of distorted shaped Mo_2C crystal, suggesting the very common phenomena among those distorted shaped flakes. All the scale bars are 5 μm.

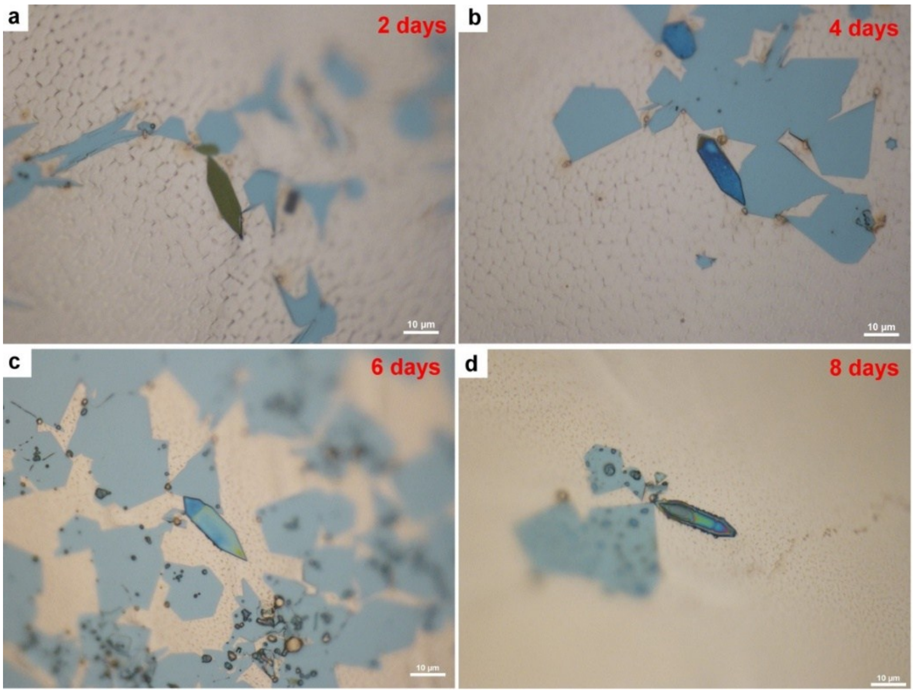

Figure S6. Surface changing of distorted hexagonal Mo_2C crystal, suggesting the very common phenomena among those distorted shaped flakes.

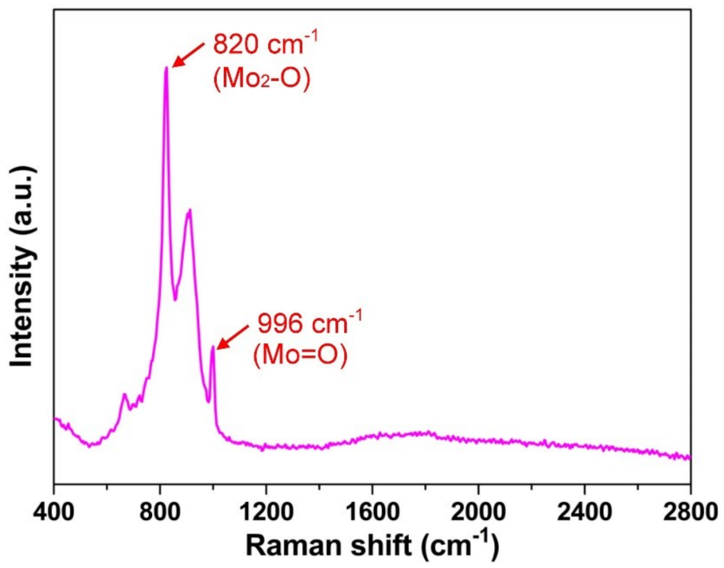

Figure S7. Raman spectrum of the as-oxidized Mo_2C samples, whereas the typical peaks for the MoO_x was labeled.

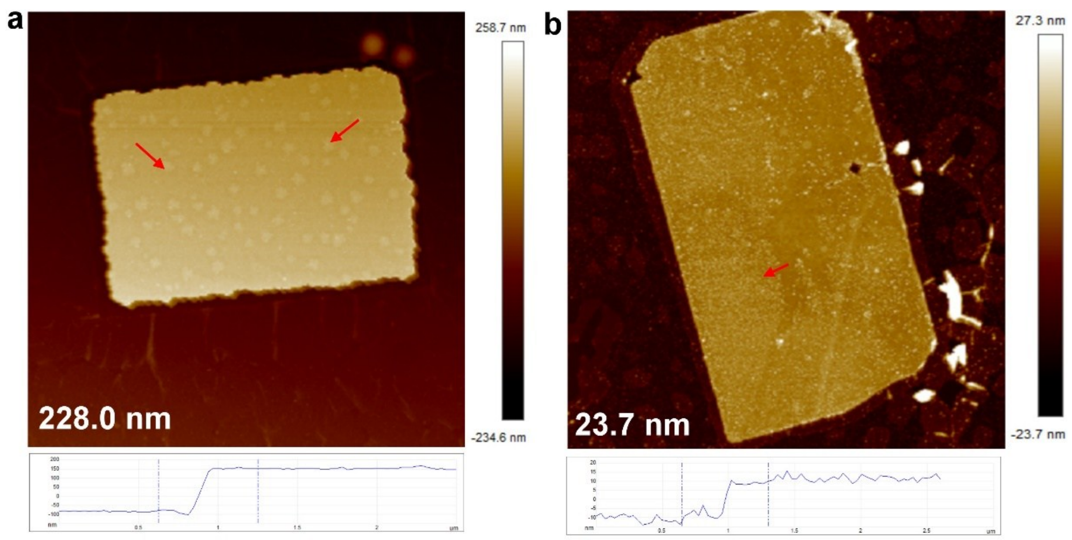

Figure S8. Thickness effect of the oxidation behavior. Noted that the surface oxidation behavior can be both detected onto the surface of elongated flakes with different thickness, indicating a common behavior.

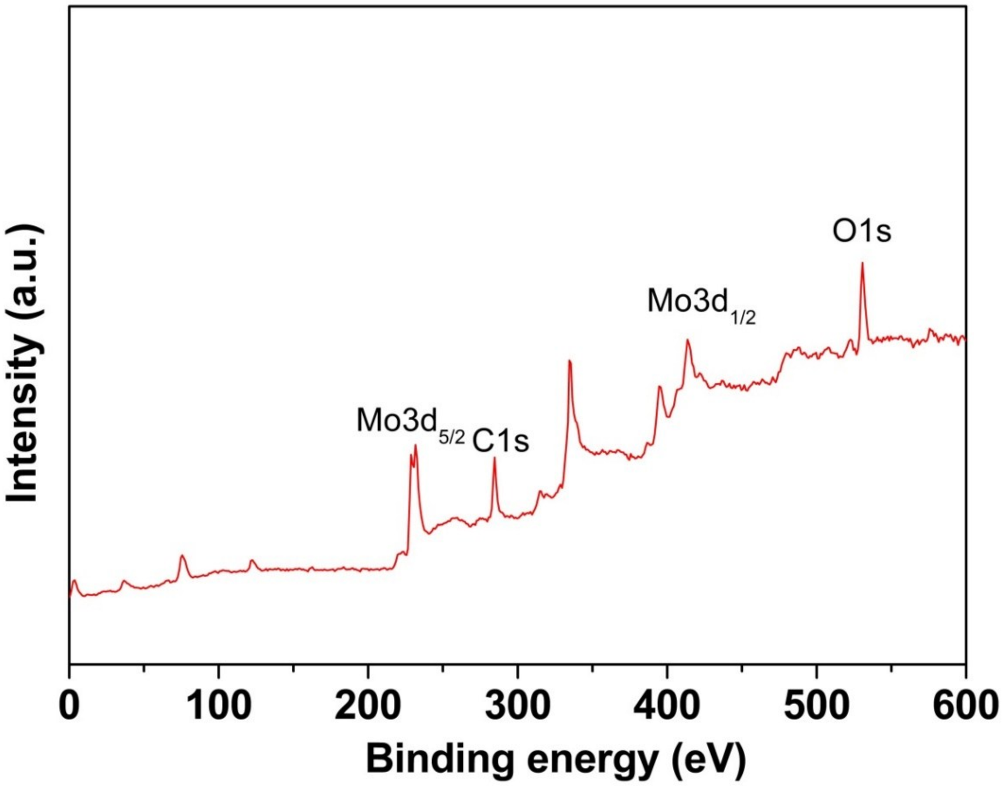

Figure S9. XPS survey spectra of the Mo_2C covered with oxides in Figure 4a.

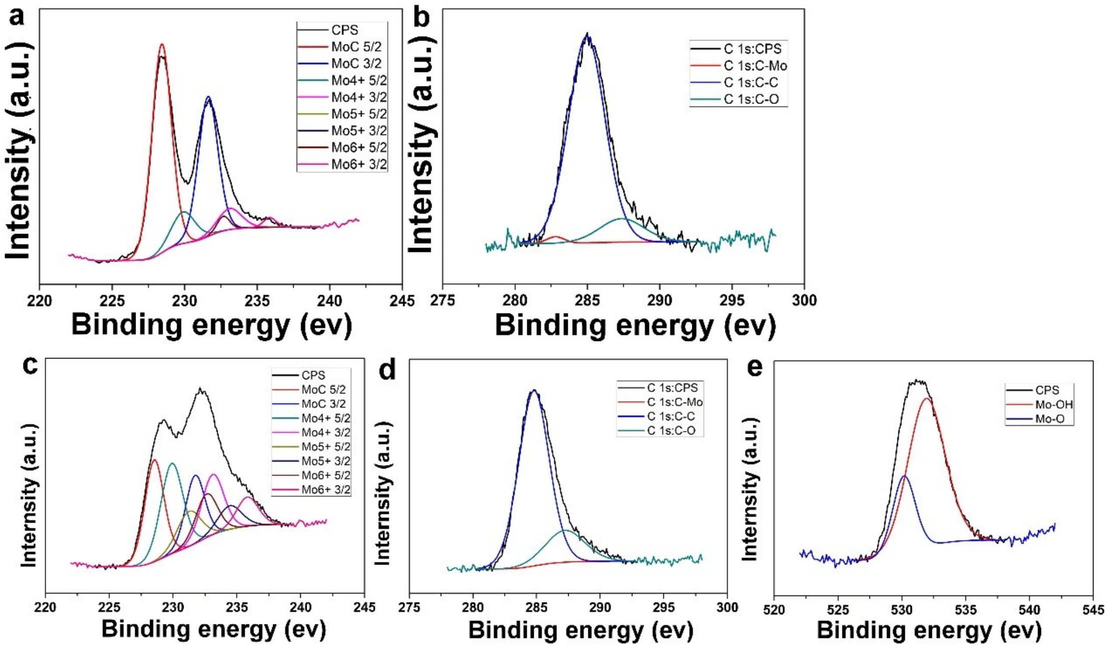

Figure S10. The XPS spectrum of before and after oxidization Mo_2C crystals.

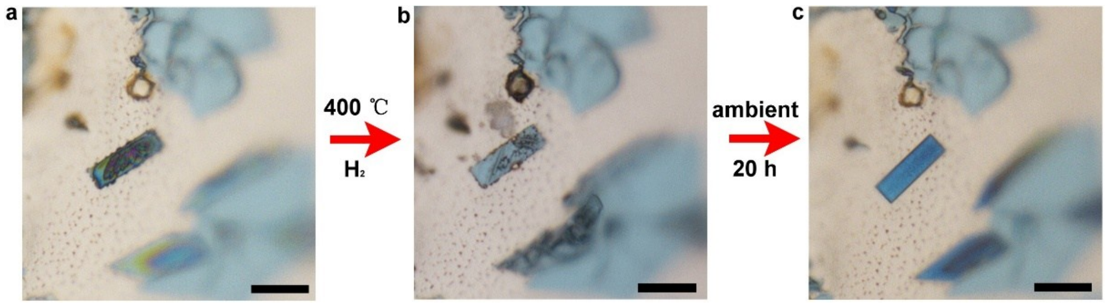

Figure S11. The reduction of the elongated Mo_2C flake on the Cu surface. All the scale bars are 10 μm.

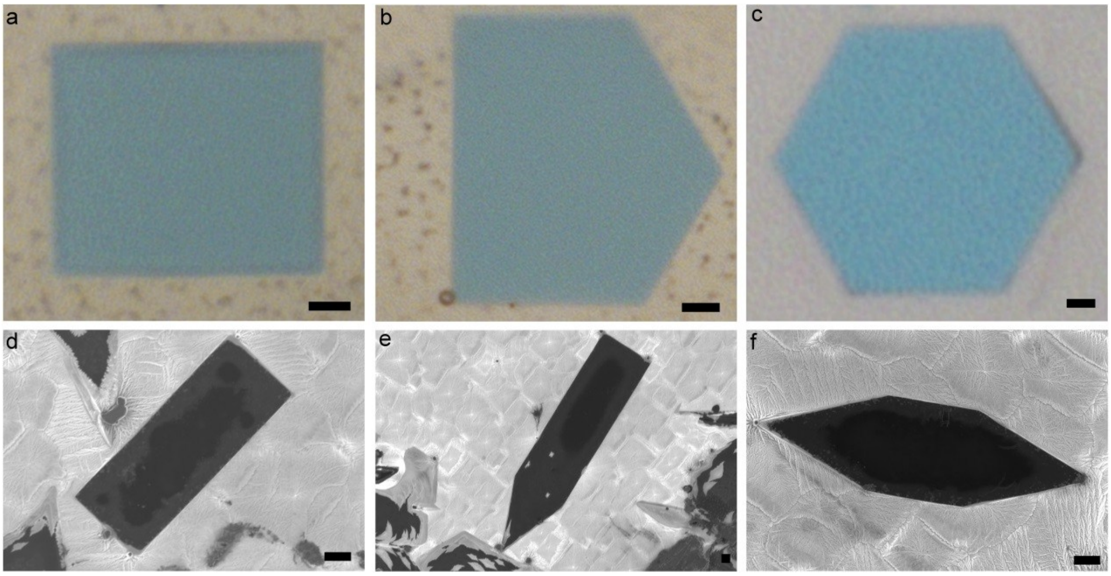

Figure S12. The regular Mo2C crystals and the corresponding elongated ones. All the scale bars are 1 μm.

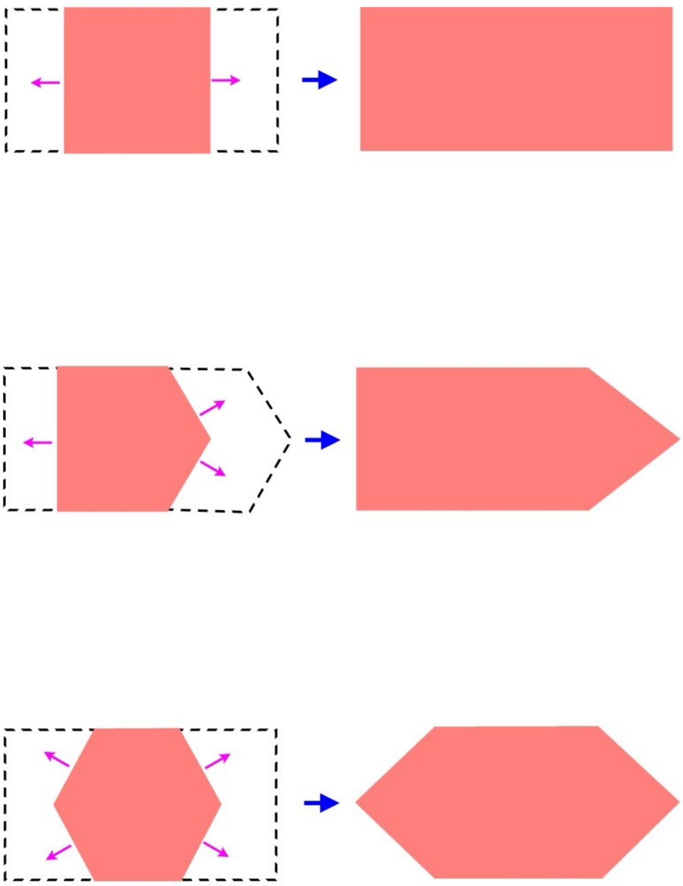

Figure S13. Schematic showing shape evolution from regular to elongated ones.

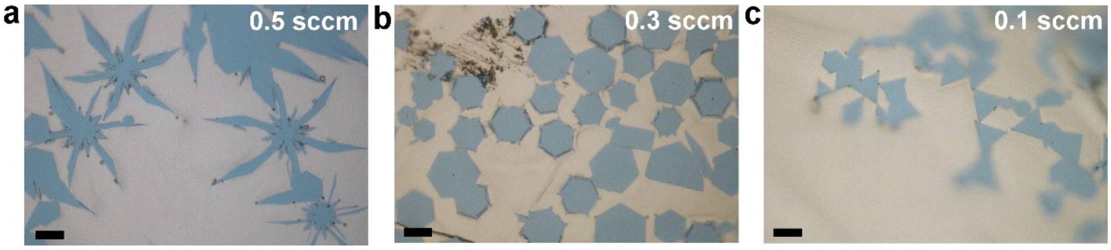

Figure S14. The as-grown Mo_2C flakes with various shapes. It is noted that the shape is sensitive to gas flow rate of CH_4. The fractal and triangular Mo_2C were obtained under certain growth conditions. All the scale bars are 5 μm.

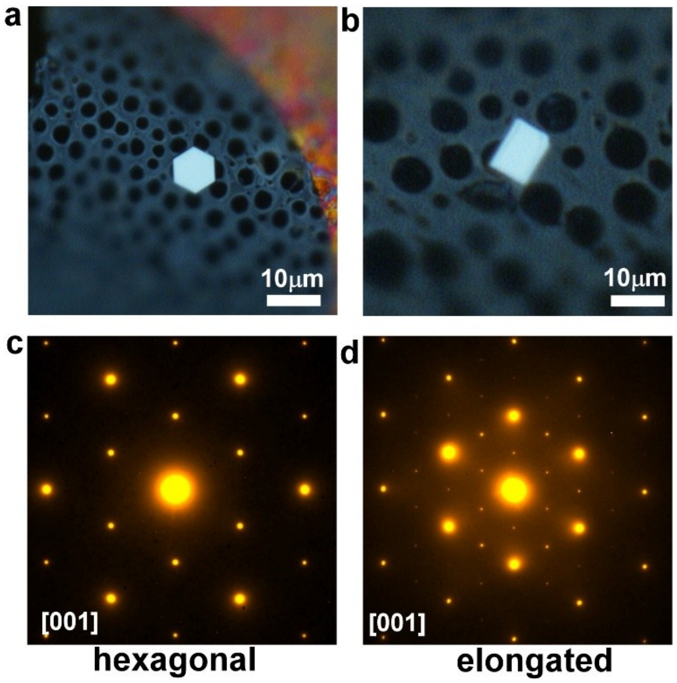

Figure S15. The TEM images and diffraction patterns of the hexagonal and elongated Mo_2C crystals, noted that the diffraction data is collected from the middle of the samples.

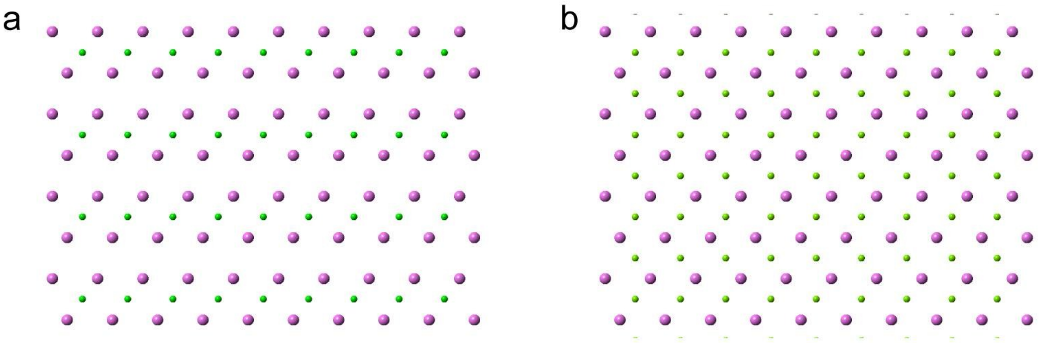

Figure S16. The side view of atomic model of regular and elongated Mo_2C crystals, respectively.

Reference

1. Baltrusaitis, J., Mendoza-Sanchez, B., Fernandez, V., Veenstra, R., Dukstiene, N., Roberts, A., & Fairley, N. (2015). Generalized molybdenum oxide surface chemical state XPS determination via informed amorphous sample model. *App. Sur. Sci.* 326, 151–161.
2. Fernandez, V., Kiani, D., Fairley, N., Felpin, F.-X., & Baltrusaitis, J. (2019). Curve

fitting complex X-ray photoelectron spectra of graphite-supported copper nanoparticles using informed line shapes. *Appl. Sur. Sci.* 143841.

3. Óvári, L., Kiss, J., Farkas, A. P., & Solymosi, F. (2005). Surface and Subsurface Oxidation of Mo2C/Mo(100): Low-Energy Ion-Scattering, Auger Electron, Angle-Resolved X-Ray Photoelectron, and Mass Spectroscopy Studies. *The Journal of Physical Chemistry B*, 109(10), 4638–4645.
4. Baltrusaitis, J., Mendoza-Sanchez, B., Fernandez, V., Veenstra, R., Dukstiene, N., Roberts, A., & Fairley, N. (2015). Generalized molybdenum oxide surface chemical state XPS determination via informed amorphous sample model. *App. Sur. Sci.* 326, 151–161.
5. Wan, C., Regmi, Y. N., & Leonard, B. M. (2014). Multiple Phases of Molybdenum Carbide as Electrocatalysts for the Hydrogen Evolution Reaction. *Angewandte Chemie International Edition* 53(25), 6407–6410.
